# The influence of music on technical skill in robotic surgery among general surgery residents: a simulation-based experimental study

**DOI:** 10.1007/s11701-026-04100-2

**Published:** 2026-11-07

**Authors:** Guilherme Corvello Dias, Ramon Jorge Guiu, Pedro Henrique Alves Silva, Heitor Zancheta de Andrade, Rodrigo Tavore Strasser, Carlos Dario da Silva Costa, Marco Antônio Ribeiro Filho, Gabriela Gouvea Silva

**Affiliations:** General Surgery Residency Program, Faculty of Medicine of São José Do Rio Preto (FAMERP), São José Do Rio Preto, SP, Brazil

**Keywords:** Music, Robotic surgery, Simulation training, Surgical education, Medical residency

## Abstract

Music is commonly played in operating rooms, but its effect on the acquisition of robotic surgical skills is unclear, particularly when the music is chosen by the trainee. This study evaluated whether a personalized musical intervention influences the technical performance of general surgery residents on a robotic surgery simulator. Thirty-two residents with no previous robotic experience performed three exercises (Pick and Place, Ring and Rail 1 and Energy Dissection 1) on the RobotiX Mentor simulator in three consecutive runs: run 1 without music, run 2 with self-selected preferred music and run 3 under a condition allocated by sealed envelope for each exercise (no music, preferred music or random music). The effect of music was assessed only in run 3, using the Kruskal–Wallis test with Bonferroni correction for 26 comparisons and epsilon-squared effect sizes. No comparison reached statistical significance after correction; the lowest unadjusted p values were observed for instrument collisions in Pick and Place (p = 0.045) and inappropriately applied energy in Energy Dissection 1 (p = 0.029). Effect sizes were mostly small, although some were moderate. Execution time decreased from run 1 to run 2 in all exercises (p ≤ 0.003), with larger reductions in more complex tasks, a pattern attributable to task repetition; because these runs were not randomized, they cannot support causal inferences about music. Under the conditions evaluated, personalized music was not associated with a significant improvement in technical performance during early robotic simulator training. Larger, fully randomized studies are warranted.

**Supplementary Information:**

The online version contains supplementary material available at https://doi.org/10.1007/s11701-026-04100-2.

## Introduction

Robotic surgery has become a major technological development in minimally invasive surgery, overcoming some of the limitations of conventional laparoscopy. Its incorporation into clinical practice has made evident the need for training methods capable of developing specific technical skills before patient care. Unlike conventional laparoscopy, robotic surgery requires adaptation to a new surgeon–platform interface, distinct visuospatial coordination and mastery of highly precise movements. In this context, robotic surgery simulators have come to play a central role in surgical training, enabling repetitive, safe and standardized practice without risk to patients [1, 2], while providing objective metrics of performance and efficiency. These measures allow training progression to be tracked and interventions that might optimize technical learning to be evaluated.

Among the environmental factors capable of influencing performance during surgical activities, music has attracted growing interest over the past decades. Its use is common in operating rooms and has been associated with an improved work environment, reduced occupational stress and increased concentration among the surgical team [3, 4]. Several studies have evaluated the impact of music on technical performance during simulated surgical tasks. Some observed improved technical efficiency, shorter execution time and lower cognitive load during laparoscopic and robotic tasks [5–9], whereas others found no significant differences between conditions with and without music [10–13].

Historically, most studies have used standardized music, such as classical music or playlists selected by the researchers. Although this strategy facilitates experimental standardization, it reduces the ecological validity of the findings, because it does not reproduce the everyday surgical environment, in which the choice of music is generally driven by the individual preferences of the team [7]. More recently, attention has turned to preferred music, on the premise that the effects of music on cognitive and motor performance depend more on familiarity, preference and the emotional meaning attributed by the individual than on the musical genre itself [7–14]. Nevertheless, studies investigating individualized musical interventions during robotic surgery training with objective simulator metrics remain scarce, particularly among general surgery residents without previous robotic experience, and the musical interventions described in the literature are poorly characterized.

The aim of this study was therefore to evaluate the influence of a personalized musical intervention on the technical performance of general surgery residents during robotic simulator training. We compared no music, the participant’s preferred music and random music using the objective metrics automatically recorded by the simulator, and characterized the musical repertoire chosen by the participants.

## Methods

### Study design and setting

This prospective experimental study was conducted at the Surgical Simulation Laboratory of the Faculty of Medicine of São José do Rio Preto (FAMERP), Brazil, between May 2025 and February 2026, using the RobotiX Mentor virtual reality simulator (Surgical Science, Gothenburg, Sweden), which records objective performance metrics during simulated surgical tasks [15]. The study was approved by the institutional Research Ethics Committee (CAAE 86407625.8.0000.5415), and all participants provided written informed consent before data collection.

### Participants

All residents enrolled in the FAMERP General Surgery Residency Program were invited, and all 32 agreed to participate, with no refusals. The only eligibility criterion was the absence of previous experience in robotic surgery; none of the participants had previous training on the simulator used in the study. No other participant characteristics were recorded.

### Simulator exercises

All participants received standardized instructions on the operation of the simulator and the execution of the exercises through the same explanatory video. No warm-up or familiarization run was performed, because the study was designed to assess participants without any previous contact with robotic simulation; consequently, run 1 also represented each participant’s first exposure to the simulator. Three exercises of increasing difficulty were used: *Pick and Place*, in which coloured pegs are transferred to the tray of the corresponding colour; *Ring and Rail 1*, in which a ring is guided along a curved metal rail while the camera is moved simultaneously; and *Energy Dissection 1*, in which a main blood vessel is isolated from its branches using energy instruments.

### Experimental protocol

Each participant performed three consecutive runs of the three exercises. The first two runs followed a fixed order: run 1 without music and run 2 with the participant’s preferred music, chosen in advance. For run 3, the condition was allocated separately for each exercise using sealed, opaque envelopes prepared by the investigators before data collection. Three sets of envelopes were prepared, one for each exercise, and each envelope contained the instruction for the final attempt (no music, preferred music or random music). Before data collection, each participant drew one envelope from each set; its content was seen only by the investigator, who then set up the corresponding condition. Allocation was neither blocked nor stratified, which resulted in unequal group sizes, and a participant could perform different exercises under different conditions. In the random music condition, the song was drawn from a separate playlist previously compiled by the investigator, containing 30 tracks of different genres (pop, Brazilian and international rock, sertanejo, MPB, pagode and funk); the participant’s preferred song and its genre were excluded from the draw, and familiarity with the drawn song was not assessed. Music was delivered through headphones at a volume chosen by each participant, without measurement of the sound pressure level, and played continuously until the end of the run, restarting whenever the run lasted longer than the song. Runs were performed consecutively, without rest intervals. Location, equipment, lighting, simulator position and instructions were kept constant throughout the experiment.

### Outcome measures

The outcomes were the objective metrics recorded automatically by the simulator software at the end of each exercise: execution time, economy of motion, workspace, instrument collisions, instruments out of view, grasper contact warnings, excessive instrument force and drops (pegs or rings), together with metrics specific to Energy Dissection 1: inappropriately applied energy, blood loss and injured vessels. Execution time, grasper contact warnings, excessive instrument force and inappropriately applied energy were recorded in seconds; economy of motion, workspace and instruments out of view in centimetres; blood loss in millilitres; and collisions, drops and injured vessels as counts. For each preferred song, the title, artist, genre, origin (Brazilian or international), presence of lyrics, perceived tempo and perceived energy were recorded. Perceived tempo and energy were reported by each participant for the song they had chosen. No objective acoustic characteristics of the songs (such as tempo in beats per minute) and no ratings of arousal, valence or familiarity were recorded.

### Statistical analysis

Analyses were performed using R version 4.4.1 [16]. Because the musical condition was confounded with run order in runs 1 and 2, the effect of music was assessed only in the randomly allocated run (run 3). For each exercise, metrics were summarized by condition as median and interquartile range (IQR), and the three conditions were compared with the Kruskal–Wallis test, a non-parametric test suitable for small samples and non-normally distributed data. A total of 26 tests were performed (8 for Pick and Place, 8 for Ring and Rail 1 and 10 for Energy Dissection 1). The Bonferroni correction was applied by multiplying each p value by the number of tests, and differences were considered significant when the corrected p value was below 0.05. Effect sizes were estimated as epsilon squared (ε2 = H/(n − 1)). Distributions of selected metrics by condition were displayed in box plots using the ggplot2 package, version 4.0.3 [17].

To characterize the learning effect, execution time in run 1 and run 2 was compared within participants for each exercise using the Wilcoxon signed-rank test, and the relative change between the two runs was calculated for each participant. Because these runs differed in both order and musical condition, this analysis describes the effect of task repetition and was not used to infer any effect of music. Descriptive statistics pooling all runs performed under each condition are provided in Online Resource 1 for transparency only.

## Results

### Participants and musical intervention

All 32 invited residents completed the protocol, and there were no missing data. In run 3, the 96 attempts comprised 27 without music, 33 with preferred music and 36 with random music (Pick and Place, 10/10/12; Ring and Rail 1, 9/12/11; Energy Dissection 1, 8/11/13, for no music/preferred/random music, respectively). The 32 participants chose 31 different songs performed by 25 artists; one song was chosen independently by two participants. Rock/alternative (9; 28.1%), sertanejo (8; 25.0%) and pop (7; 21.9%) were the most frequent genres; 20 songs (62.5%) were international and 12 (37.5%) Brazilian. All songs had lyrics, and most were perceived by the participants as moderate in tempo (24; 75.0%) and energy (24; 75.0%) (Table 1). The full catalogue of songs is available in Online Resource 1.

**Table 1:** Characteristics of the personalized musical intervention (preferred songs chosen by the 32 participants)

Characteristic	n (%)
Different songs	31
Different artists	25
**Genre**	
Rock/alternative	9 (28.1)
Sertanejo	8 (25.0)
Pop	7 (21.9)
MPB	3 (9.4)
Rap/hip-hop	2 (6.3)
Indie/folk	2 (6.3)
Brazilian rock	1 (3.1)
**Origin**	
International	20 (62.5)
Brazilian	12 (37.5)
**Lyrics**	
Yes	32 (100.0)
**Perceived tempo**	
Moderate	24 (75.0)
Fast	6 (18.8)
Slow	2 (6.3)
**Perceived energy**	
Moderate	24 (75.0)
High	8 (25.0)

MPB, *música popular brasileira* (Brazilian popular music). Percentages refer to the 32 participants; one song was chosen by two participants. The full catalogue is provided in Online Resource 1

### Comparison between conditions in the randomized run

**Fig. 1:**
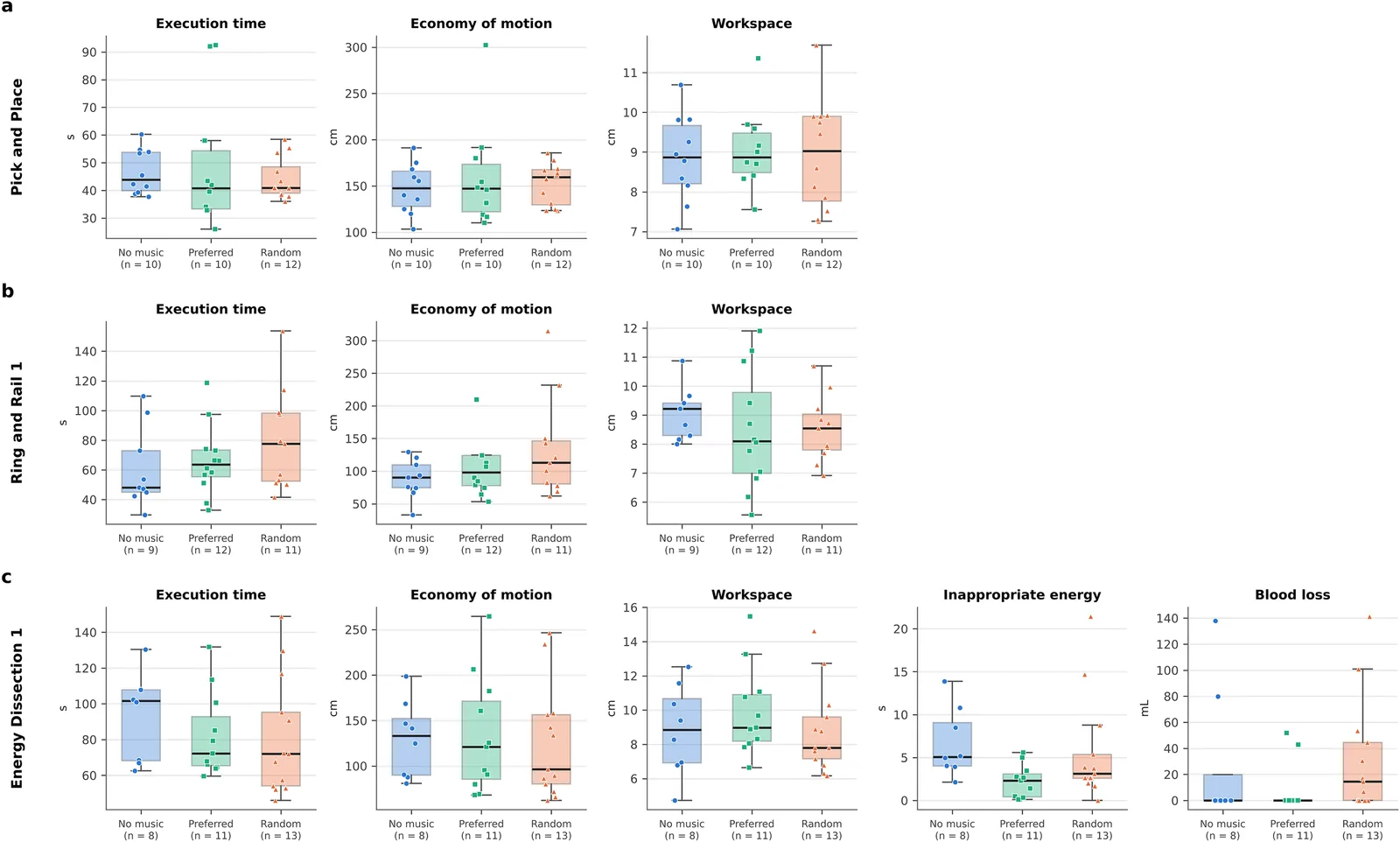
Distribution of selected simulator metrics by condition in the randomly allocated run (run 3): **a** Pick and Place, **b** Ring and Rail 1 and **c** Energy Dissection 1. Points represent individual attempts; boxes show the median and interquartile range; whiskers extend to the most extreme values within 1.5 times the interquartile range

Table 2 shows the median values of each metric by condition in run 3. None of the 26 comparisons reached statistical significance after Bonferroni correction. The lowest unadjusted p values were observed for instrument collisions in Pick and Place (H = 6.19, p = 0.045; corrected p = 1.000) and inappropriately applied energy in Energy Dissection 1 (H = 7.08, p = 0.029; corrected p = 0.753). Because no test was significant, no post hoc comparisons were performed. Effect sizes were small for most comparisons (ε2 < 0.08 in 20 of 26 tests) but were 0.08 or higher for six metrics, the largest being inappropriately applied energy in Energy Dissection 1 (ε2 = 0.23) and instrument collisions in Pick and Place (ε2 = 0.20). The distributions of the selected metrics overlapped considerably among the three conditions (Fig. 1).

**Table 2:** Simulator metrics by condition in the randomly allocated run (run 3) and Kruskal–Wallis comparison

Metric	No music	Preferred music	Random music	H	p	p (Bonf.)	ε^2^
**Pick and Place (n = 10/10/12)**							
Execution time (s)	43.88 [39.88–53.81]	40.79 [33.34–54.31]	40.94 [38.99–48.55]	0.73	0.693	1.000	0.024
Economy of motion (cm)	147.79 [127.83–166.01]	147.22 [122.16–173.59]	159.61 [129.74–167.57]	0.26	0.880	1.000	0.008
Workspace (cm)	8.86 [8.21–9.67]	8.87 [8.48–9.48]	9.03 [7.77–9.90]	0.04	0.982	1.000	0.001
Instrument collisions (n)	0.00 [0.00–1.00]	0.00 [0.00–0.00]	0.00 [0.00–0.00]	6.19	0.045	1.000	0.200
Instruments out of view (cm)	0.00 [0.00–0.00]	0.00 [0.00–0.00]	0.00 [0.00–0.00]	1.67	0.435	1.000	0.054
Grasper contact warnings (s)	0.00 [0.00–0.00]	0.00 [0.00–0.24]	0.00 [0.00–0.22]	1.32	0.516	1.000	0.043
Excessive instrument force (s)	0.00 [0.00–0.00]	0.00 [0.00–0.00]	0.00 [0.00–0.00]	0.41	0.814	1.000	0.013
Drops (n)	0.00 [0.00–0.75]	0.00 [0.00–0.75]	0.00 [0.00–0.00]	0.59	0.743	1.000	0.019
**Ring and Rail 1 (n = 9/12/11)**							
Execution time (s)	48.09 [45.00–72.93]	63.59 [55.28–73.31]	77.71 [52.30–98.16]	2.35	0.309	1.000	0.076
Economy of motion (cm)	90.17 [74.30–109.86]	98.23 [77.64–124.25]	113.19 [80.33–146.48]	1.86	0.395	1.000	0.060
Workspace (cm)	9.22 [8.30–9.41]	8.11 [6.99–9.78]	8.55 [7.79–9.04]	1.87	0.394	1.000	0.060
Instrument collisions (n)	0.00 [0.00–0.00]	0.00 [0.00–0.31]	1.00 [0.00–2.00]	4.76	0.092	1.000	0.154
Instruments out of view (cm)	1.18 [0.00–1.83]	0.00 [0.00–2.90]	0.06 [0.00–2.88]	0.29	0.867	1.000	0.009
Grasper contact warnings (s)	0.00 [0.00–0.00]	0.00 [0.00–4.09]	0.00 [0.00–0.00]	2.30	0.317	1.000	0.074
Excessive instrument force (s)	0.43 [0.00–1.02]	0.00 [0.00–1.43]	0.88 [0.00–1.44]	0.80	0.669	1.000	0.026
Drops (n)	0.00 [0.00–0.00]	0.00 [0.00–0.00]	0.00 [0.00–0.00]	2.56	0.279	1.000	0.082
**Energy Dissection 1 (n = 8/11/13)**							
Execution time (s)	101.68 [68.19–107.88]	72.28 [65.31–92.88]	72.12 [54.10–95.34]	1.69	0.430	1.000	0.054
Economy of motion (cm)	133.20 [89.72–152.11]	121.12 [85.26–171.43]	96.55 [80.16–156.30]	0.44	0.802	1.000	0.014
Workspace (cm)	8.84 [6.91–10.66]	8.97 [8.18–10.91]	7.80 [7.15–9.59]	2.01	0.366	1.000	0.065
Instrument collisions (n)	1.00 [0.00–1.50]	0.00 [0.00–1.00]	0.00 [0.00–1.00]	1.99	0.369	1.000	0.064
Instruments out of view (cm)	0.00 [0.00–0.34]	0.00 [0.00–0.96]	0.00 [0.00–0.00]	0.10	0.953	1.000	0.003
Grasper contact warnings (s)	0.00 [0.00–0.09]	0.02 [0.00–0.97]	0.55 [0.00–3.13]	1.80	0.407	1.000	0.058
Excessive instrument force (s)	0.00 [0.00–1.21]	0.00 [0.00–0.76]	0.00 [0.00–2.01]	0.06	0.971	1.000	0.002
Inappropriately applied energy (s)	5.06 [4.01–9.06]	2.32 [0.40–3.10]	3.13 [2.58–5.35]	7.08	0.029	0.753	0.229
Blood loss (mL)	0.00 [0.00–19.95]	0.00 [0.00–0.00]	14.50 [0.00–44.50]	4.06	0.131	1.000	0.131
Injured vessels (n)	0.00 [0.00–0.00]	0.00 [0.00–0.00]	0.00 [0.00–1.00]	4.02	0.134	1.000	0.130

Values are median [interquartile range]; n, number of attempts (no music/preferred/random music). Bonferroni-corrected p values were obtained by multiplying the unadjusted p value by the number of tests (26) and truncating at 1.000. ε^2^, epsilon squared (H/(n − 1)), n = 32 per exercise; df = 2 for all tests. Units: s, seconds; cm, centimetres; mL, millilitres; n, count

### Learning effect across the non-randomized runs

Median execution time decreased from run 1 to run 2 in Pick and Place (52.78 vs 45.91 s; p < 0.001), Ring and Rail 1 (88.10 vs 73.11 s; p = 0.003) and Energy Dissection 1 (121.43 vs 91.74 s; p < 0.001), with 25, 24 and 28 of the 32 residents, respectively, performing faster in run 2. The median relative reduction increased with task complexity: 11.4% (IQR 3.6% to 32.3%) in Pick and Place, 20.7% (IQR −1.5% to 46.7%) in Ring and Rail 1 and 32.4% (IQR 17.1% to 41.2%) in Energy Dissection 1. These differences reflect task repetition and cannot be attributed to music.

## Discussion

In this study, we evaluated the influence of different musical conditions on the technical performance of general surgery residents during robotic simulator training. Unlike part of the literature, which investigated surgical performance using standardized music or genres defined by the researchers, such as classical, arousing or calming music [11, 18, 19], we included a condition in which each participant trained while listening to their own preferred music. Despite this approach, no statistically significant differences were observed among no music, preferred music and random music in the randomized run after correction for multiple comparisons.

These results are in line with part of the available literature. Systematic reviews show that, although some studies have identified favourable effects of music on certain components of surgical performance, the findings remain heterogeneous and do not establish a uniform benefit of musical intervention. Differences in experimental design, participant experience, task type and complexity, characteristics of the musical stimulus and exposure conditions are potential sources of this variability [7, 20] and limit direct comparison between studies.

Among studies with favourable results, preferred music has received particular attention. In laparoscopic simulation, music chosen by the participants was associated with significantly shorter execution time, shorter instrument path length and lower mental workload [8]. In robotic surgery, differences in performance according to the type of music were also observed, with shorter execution time and instrument path length under certain musical conditions [5]. However, these effects were not uniform across stimuli and tasks, suggesting that musical characteristics, individual preference and task complexity may modulate the influence of music on performance [5, 8].

Conversely, other experimental studies have not demonstrated a consistent technical benefit of music. In a virtual reality laparoscopic environment, no significant improvement attributable to music was observed, and changes in performance were related mainly to task repetition; arousing music was even associated with worse performance in the initial assessment, suggesting that musical stimulation may act as a distractor during the acquisition of new skills [11]. More recent results have also shown a lack of consistent benefit of music in certain simulation scenarios [13]. Notably, in a randomized crossover trial with 97 novices, preferred music reduced mental workload but did not improve laparoscopic task performance [14]. These findings agree with ours and reinforce that possible psychological or physiological effects of music do not necessarily translate into measurable improvement in objective performance metrics.

The detailed characterization of the musical intervention allows a broader interpretation of these results. The 32 participants chose 31 different songs by 25 artists, spanning genres such as rock, sertanejo, pop, *música popular brasileira* (MPB) and rap; the only repeated choice, a song selected independently by two participants, reflects shared preferences within a culturally homogeneous group rather than a meaningful clustering of the intervention. This diversity shows that the intervention was based on individual preference rather than on the characteristics of a single genre, in contrast with most previous studies, which used standardized interventions, often classical music, while fewer evaluated songs chosen by the participants [7, 8]. Although preferred music has been associated with reduced mental workload and situational stress during simulated surgical tasks [8], in the present study this strategy did not translate into a significant improvement in technical performance. If music does influence performance in robotic simulation, its effect probably depends on the interaction of multiple factors rather than solely on exposure to a song the participant finds pleasant [5, 8].

The clearest finding of this study was the reduction in execution time between the first two runs. Because no familiarization run was performed, run 1 represented the participants’ first contact with the simulator, and the improvement observed in run 2 is best explained by task repetition and adaptation to the platform rather than by music, as also suggested by the absence of differences among conditions in the randomized run. The magnitude of this improvement increased with task complexity, from 11% in Pick and Place to 32% in Energy Dissection 1, a gradient consistent with motor learning and with previous reports of rapid initial learning in simulated surgical training [11, 12]. In novices, therefore, early performance gains on a robotic simulator are dominated by repetition, which may mask or mimic the effect of environmental interventions such as music.

The composition of the sample should also be considered. All participants were residents without previous robotic experience, which provided homogeneity in baseline experience and reduced the influence of previously acquired robotic skills on the metrics. Previous investigations have similarly restricted their samples to novices or adopted criteria to minimize the influence of different skill levels [5, 7, 11]. In addition, the use of a commercial simulator that provides objective, standardized metrics reduced reliance on subjective assessment. The RobotiX Mentor has been validated for the assessment and training of robotic skills [1, 2], and quantitative parameters from simulators or motion analysis are widely used to assess simulated surgical performance [7].

From a practical standpoint, these findings suggest that music need not be either encouraged or restricted during early robotic simulation training: residents may train with or without their preferred music without a measurable change in technical performance. More importantly, they show that training programmes and studies evaluating environmental interventions should account for the initial learning curve, for example by including a familiarization phase and by randomizing or counterbalancing the order of conditions, because early gains from repetition can easily be mistaken for an intervention effect. Whether music provides benefits in other training settings, such as more experienced trainees, longer training sessions or more complex procedures, remains to be determined in larger studies.

### Limitations

This study has limitations. First, only the third run was randomized, and no familiarization run was performed; because no music always corresponded to the participant’s first exposure to the simulator and preferred music always followed it, the non-randomized runs cannot support any causal inference about music. Second, the randomized comparison relied on small subgroups of 8 to 13 attempts per condition in each exercise, without an a priori sample size calculation; the study was therefore underpowered to detect small effects, and the moderate effect sizes observed for some metrics indicate that differences of practical relevance cannot be excluded. The Bonferroni correction across 26 tests, although protective against false-positive findings, further reduced power. Third, the musical stimulus was characterized only by participant-reported tempo and energy: objective acoustic variables (such as tempo in beats per minute), sound pressure level at the ear, ratings of arousal and valence, familiarity with the random songs and recent exposure to the chosen songs were not recorded, and the volume was chosen by each participant, which may itself have influenced performance. Fourth, subjective outcomes such as anxiety, cognitive load, mental fatigue and perceived performance were not assessed, so psychological effects of music unaccompanied by changes in technical metrics cannot be excluded. Fifth, apart from the absence of previous robotic experience, participant characteristics such as postgraduate year, video game experience or musical training were not recorded. Finally, this was a single-centre study of Portuguese-speaking novices performing basic tasks, whose preferred repertoire was dominated by Brazilian and locally familiar music. Because familiarity, lyrical comprehension and the emotional meaning of music depend on linguistic and cultural background, the present null result may not replicate in trainees from other linguistic or musical cultures, in experienced surgeons or in complex procedures, and multicentre, international studies would be required before these conclusions are extended to other populations.

Future studies should use fully randomized or counterbalanced sequences of conditions, a preparation phase to stabilize the initial learning curve before testing [14], objective characterization of the musical stimulus (tempo, sound pressure level, arousal, valence and familiarity), larger multicentre samples from different cultural backgrounds, participants with different levels of experience, tasks of varying complexity and simultaneous assessment of technical, psychological and physiological parameters. This would make it possible to determine whether subjective effects of music, such as reduced stress or mental workload, occur in the absence of detectable changes in technical performance, and which individual, musical or task-related characteristics modulate this response.

## Conclusion

Under the conditions evaluated, a personalized musical intervention was not associated with a statistically significant improvement in technical performance in the randomized comparison of robotic simulator runs among general surgery residents. The reduction in execution time between the first two runs reflects task repetition, and because these runs were not randomized, they cannot support any causal claim about the effect of music. Personalized musical interventions proved feasible and can be characterized in detail, providing a methodological basis for larger, fully randomized studies that include a familiarization phase and integrate technical, physiological and cognitive outcomes.

## Supplementary Information

Below is the link to the electronic supplementary material.

Supplementary file1 (PDF 189 KB)

## Data Availability

The datasets generated and analysed during the current study are available from the corresponding author on reasonable request.
