# Supplementary material for: The influence of music on technical skill in robotic surgery among general surgery residents: a simulation-based experimental study
Source: J Robot Surg. 2026 Nov 7;20(1):1181. doi: 10.1007/s11701-026-04100-2 (PMC13743583; doi:10.1007/s11701-026-04100-2)
Supplement: Supplementary file 1 [file 11701_2026_4100_MOESM1_ESM.pdf]

Online Resource 1

The influence of music on technical skill in robotic surgery among general surgery residents: a simulation-based experimental study

Journal of Robotic Surgery

Guilherme Corvello Dias, Ramon Jorge Guiu, Pedro Henrique Alves Silva, Heitor Zancheta de Andrade, Rodrigo Tavore Strasser, Carlos Dario da Silva Costa, Marco Antônio Ribeiro Filho, Gabriela Gouvea Silva

Corresponding author: Guilherme Corvello Dias, Faculty of Medicine of São José do Rio Preto (FAMERP), São José do Rio Preto, SP, Brazil; gui.corvello@gmail.com

Table S1 Catalogue of the preferred songs chosen by each participant

Participant	Song	Artist	Genre	Origin	Lyrics	Perceived tempo	Perceived energy
P01	I Wonder	Kanye West	Rap/hip-hop	International	Yes	Moderate	High
P02	Stay	Post Malone	Pop	International	Yes	Moderate	Moderate
P03	Back to Friends	sombr	Indie/folk	International	Yes	Moderate	Moderate
P04	Graveto	Marília Mendonça	Sertanejo	Brazilian	Yes	Moderate	Moderate
P05	Yellow	Coldplay	Rock/alternative	International	Yes	Slow	Moderate
P06	Iris	The Goo Goo Dolls	Rock/alternative	International	Yes	Moderate	Moderate
P07	Otherside	Red Hot Chili Peppers	Rock/alternative	International	Yes	Moderate	Moderate
P08	All Star	Nando Reis	MPB	Brazilian	Yes	Moderate	Moderate
P09	Eye of the Tiger	Survivor	Rock/alternative	International	Yes	Fast	High
P10	Crazy	Aerosmith	Rock/alternative	International	Yes	Fast	High
P11	Unwritten	Natasha Bedingfield	Pop	International	Yes	Fast	High
P12	The Fate of Ophelia	Taylor Swift	Pop	International	Yes	Moderate	Moderate
P13	Under the Bridge	Red Hot Chili Peppers	Rock/alternative	International	Yes	Moderate	Moderate
P14	Mil Anos	Jorge & Mateus	Sertanejo	Brazilian	Yes	Moderate	Moderate
P15	Dormi na Praça	Bruno & Marrone	Sertanejo	Brazilian	Yes	Moderate	Moderate
P16	Cruel Summer	Taylor Swift	Pop	International	Yes	Fast	High
P17	Última Saudade	Henrique & Juliano	Sertanejo	Brazilian	Yes	Moderate	Moderate
P18	Lugar ao Sol	Charlie Brown Jr.	Brazilian rock	Brazilian	Yes	Moderate	Moderate
P19	Antônimos	Jorge & Mateus	Sertanejo	Brazilian	Yes	Moderate	Moderate
P20	Ordinary	Alex Warren	Pop	International	Yes	Moderate	Moderate

Participant	Song	Artist	Genre	Origin	Lyrics	Perceived tempo	Perceived energy
P21	Prisão sem Grades	Jorge & Mateus	Sertanejo	Brazilian	Yes	Moderate	Moderate
P22	The Wicker Man	Iron Maiden	Rock/alternative	International	Yes	Fast	High
P23	Leão	Marília Mendonça	Sertanejo	Brazilian	Yes	Moderate	Moderate
P24	Till I Collapse	Eminem feat. Nate Dogg	Rap/hip-hop	International	Yes	Fast	High
P25	Feijoada Completa	Chico Buarque	MPB	Brazilian	Yes	Moderate	Moderate
P26	Wish You Were Here	Pink Floyd	Rock/alternative	International	Yes	Slow	Moderate
P27	Bohemian Rhapsody	Queen	Rock/alternative	International	Yes	Moderate	High
P28	Man Down	Rihanna	Pop	International	Yes	Moderate	Moderate
P29	Stolen Dance	Milky Chance	Indie/folk	International	Yes	Moderate	Moderate
P30	Pode Se Achegar	Tiago Iorc feat. Agnes Nunes	MPB	Brazilian	Yes	Moderate	Moderate
P31	O Mundo É Tão Pequeno	Jorge & Mateus	Sertanejo	Brazilian	Yes	Moderate	Moderate
P32	Ordinary	Alex Warren	Pop	International	Yes	Moderate	Moderate

MPB, *música popular brasileira* (Brazilian popular music)

Table S2 Simulator metrics by condition pooling all runs performed under each condition (provided for transparency only)

Metric	No music	Preferred music	Random music
**Pick and Place**			
Execution time (s)	58.45 (22.98)<br>52.69 [30.84–138.41]	48.96 (16.40)<br>43.64 [25.99–92.47]	44.33 (7.52)<br>40.94 [36.07–58.49]
Economy of motion (cm)	165.63 (50.52)<br>155.45 [103.32–354.22]	157.31 (41.52)<br>147.22 [104.12–302.24]	152.43 (22.32)<br>159.61 [123.36–185.94]
Workspace (cm)	9.08 (1.49)<br>8.98 [6.85–14.17]	9.13 (1.23)<br>8.88 [7.32–11.91]	8.94 (1.37)<br>9.03 [7.26–11.69]
Instrument collisions (n)	0.69 (1.33)<br>0.00 [0.00–6.00]	0.43 (1.06)<br>0.00 [0.00–4.00]	0.00 (0.00)<br>0.00 [0.00–0.00]
Instruments out of view (cm)	0.41 (2.68)<br>0.00 [0.00–17.34]	0.00 (0.00)<br>0.00 [0.00–0.00]	0.06 (0.22)<br>0.00 [0.00–0.77]
Grasper contact warnings (s)	1.36 (3.60)<br>0.00 [0.00–19.39]	0.69 (1.69)<br>0.00 [0.00–8.24]	0.29 (0.54)<br>0.00 [0.00–1.59]
Excessive instrument force (s)	0.11 (0.39)<br>0.00 [0.00–2.07]	0.07 (0.23)<br>0.00 [0.00–1.35]	0.04 (0.09)<br>0.00 [0.00–0.27]
Drops (n)	0.43 (0.80)<br>0.00 [0.00–3.00]	0.38 (0.91)<br>0.00 [0.00–5.00]	0.25 (0.62)<br>0.00 [0.00–2.00]
**Ring and Rail 1**			
Execution time (s)	101.34 (59.63)<br>72.93 [29.74–264.25]	74.21 (31.14)<br>67.33 [32.92–172.96]	79.51 (34.20)<br>77.71 [41.51–153.78]
Economy of motion (cm)	152.28 (112.45)<br>120.39 [33.11–616.22]	119.22 (62.07)<br>106.49 [46.46–330.11]	133.26 (77.14)<br>113.19 [61.73–314.97]
Workspace (cm)	9.59 (1.76)<br>9.41 [7.03–15.51]	8.96 (1.64)<br>8.98 [5.56–14.01]	8.52 (1.14)<br>8.55 [6.91–10.70]
Instrument collisions (n)	1.41 (3.56)<br>0.00 [0.00–18.00]	0.73 (1.23)<br>0.00 [0.00–5.00]	1.00 (0.89)<br>1.00 [0.00–2.00]
Instruments out of view (cm)	7.29 (18.78)<br>1.35 [0.00–95.21]	3.00 (5.06)<br>0.34 [0.00–23.28]	2.80 (4.76)<br>0.06 [0.00–12.20]
Grasper contact warnings (s)	0.91 (2.67)<br>0.00 [0.00–15.57]	1.62 (3.28)<br>0.00 [0.00–15.79]	0.07 (0.20)<br>0.00 [0.00–0.67]

Metric	No music	Preferred music	Random music
Excessive instrument force (s)	2.75 (9.00)<br>0.78 [0.00–55.46]	1.53 (4.62)<br>0.39 [0.00–30.07]	1.25 (1.82)<br>0.88 [0.00–6.00]
Drops (n)	0.44 (0.87)<br>0.00 [0.00–3.00]	0.18 (0.50)<br>0.00 [0.00–2.00]	0.00 (0.00)<br>0.00 [0.00–0.00]
**Energy Dissection 1**			
Execution time (s)	126.04 (45.90)<br>107.91 [62.58–249.60]	95.86 (48.99)<br>83.95 [55.83–295.96]	81.24 (32.93)<br>72.12 [45.96–148.88]
Economy of motion (cm)	179.49 (70.65)<br>176.07 [80.81–336.60]	151.56 (104.76)<br>120.97 [62.61–608.55]	124.90 (61.20)<br>96.55 [62.11–246.54]
Workspace (cm)	9.32 (2.16)<br>9.25 [4.72–17.45]	9.45 (2.48)<br>8.87 [5.90–16.92]	8.81 (2.51)<br>7.80 [6.16–14.63]
Instrument collisions (n)	1.38 (2.71)<br>0.00 [0.00–14.00]	0.79 (1.57)<br>0.00 [0.00–7.00]	0.85 (1.77)<br>0.00 [0.00–6.00]
Instruments out of view (cm)	3.96 (10.51)<br>0.00 [0.00–57.92]	9.65 (47.78)<br>0.00 [0.00–312.03]	2.54 (6.98)<br>0.00 [0.00–25.00]
Grasper contact warnings (s)	2.72 (6.90)<br>0.00 [0.00–35.25]	3.17 (6.63)<br>0.00 [0.00–27.24]	3.83 (9.78)<br>0.55 [0.00–35.88]
Excessive instrument force (s)	4.91 (11.63)<br>0.00 [0.00–45.10]	6.62 (22.91)<br>0.00 [0.00–141.01]	3.46 (9.29)<br>0.00 [0.00–34.00]
Inappropriately applied energy (s)	6.55 (5.29)<br>4.95 [0.00–18.87]	4.18 (4.34)<br>2.76 [0.00–21.92]	5.59 (6.07)<br>3.13 [0.00–21.44]
Blood loss (mL)	70.98 (91.68)<br>29.00 [0.00–333.90]	58.09 (139.67)<br>0.00 [0.00–762.90]	31.48 (44.36)<br>14.50 [0.00–141.30]
Injured vessels (n)	0.33 (0.73)<br>0.00 [0.00–3.00]	0.35 (0.72)<br>0.00 [0.00–3.00]	0.46 (0.66)<br>0.00 [0.00–2.00]

Values are mean (SD) and median [minimum–maximum]. No music comprises run 1 (plus run 3 when allocated), preferred music comprises run 2 (plus run 3 when allocated) and random music comprises run 3 only. Because condition is confounded with run order and learning, these data should not be used to compare conditions. Number of attempts (no music/preferred/random music): Pick and Place, 42/42/12; Ring and Rail 1, 41/44/11; Energy Dissection 1, 40/43/13
